# Integral Valorization of Pineapple (*Ananas comosus* L.) By-Products through a Green Chemistry Approach towards Added Value Ingredients

**DOI:** 10.3390/foods9010060

**Published:** 2020-01-07

**Authors:** Débora A. Campos, Tânia B. Ribeiro, José A. Teixeira, Lorenzo Pastrana, Maria Manuela Pintado

**Affiliations:** 1 CBQF–Centro de Biotecnologia e Química Fina–Laboratório Associado, Escola Superior de Biotecnologia, Univerisdade Católica Portuguesa, Rua Diogo Botelho 1327, 4169-005 Porto, Portugal; dcampos@porto.ucp.pt (D.A.C.); tania.ribeiro@blc3.pt (T.B.R.); 2 Centro de Engenharia Biológica, Universidade do Minho, Campus Gualtar, 4710-057 Braga, Portugal; jateixeira@deb.uminho.pt; 3 INL–International Iberian Nanotechnology Laboratory, 4710-330 Braga, Portugal; lorenzo.pastrana@inl.int

**Keywords:** pineapple by-products, green chemistry approach, integral valorization, pineapple juices, pineapple stem and peel juices

## Abstract

Industrial by-products are produced every day through fruit processing industries. Pineapple is not an exception; when processed, around 60% (*w/w*) of its weight are peels, stem, trimmings, and crown, the only used fruit part for human consumption. Due to high concerns of sustainability in the food system and negative high impact of human practice in the environment, a strategy has to be developed. Therefore, a green chemistry approach was applied to pineapple by-products to make an integrated valorization by the extraction of bioactive molecules. Two pineapple by-products (peels and stems) were studied, applying a green chemistry approach, which means the non-use of organic solvents or extreme methodologies. A subdivision of each by-product was done by the application of a juice machine. The peels and stems in the fresh state were ground separately, creating two fractions for each by-product—a juice and a wet pulp (press cake). The press cake was characterized, dried, and ground to create a fine powder flour. To the juice, a precipitation methodology with polysaccharides was applied, which allowed the bromelain separation (developing of an enzymatic fraction) from the fruit juice. The enzymatic extract was freeze-dried, and the juice was spray-dried, developing two more fine powders. Thus, three new ingredients were produced from each by-product, creating a total of six new ingredients. Overall, the enzymatic fractions represented around 0.26% (*w/w*) of pineapple weight. Pineapple stem juice represented 4.8% (*w/w*), and peel juice represented 17.3% (*w/w*). Pineapple stem flour represented 3.1% (*w/w*), and peel flour represented 11.4% (*w/w*) of the total pineapple weight. To valorize the by-products juices, a full characterization was performed of bioactive molecules and biological activities. When comparing the two juices, the peel juice showed lower content of total phenolic compounds, lower antioxidant capacity, and lower content of vitamin C. The different phenolic compounds were identified by HPLC analysis in the two pineapple by-products juices. However, the same compounds in both juices were quantified (chlorogenic, caffeic, and ferulic acids). On the other hand, the by-products flours had a high content of insoluble dietary fiber (IDF), mainly cellulose and hemicellulose. Therefore, the approach applied in this work opens the door to the production of green products, as a result of by-products valorization. This could be applied not only in the food industry but also in the nutraceutical and cosmetic industries.

## 1. Introduction

Pineapple fruits (*Ananas comosus* L.) are grown extensively in several parts of the world, being the most important the Caribbean, Asia (Malaysia and Thailand), and Africa (South Africa and Kenya). The most commercialized variety of pineapple is “Smooth Cayenne” and can be consumed directly or used for processing. Several products are nowadays commercialized, such as canned fruit, juice concentrates, jams, crystallized fruit, and dehydrated snacks. Pineapple is also one of the primary ingredients used as the basis for other fruit concentrates due to its neutral color and flavor. Currently, it is one of the most exported fruits in the world, representing a substantial economic impact on trading among several countries. Throughout the pineapple production and consumption chain, several tons of by-products are produced, which, in most cases, are discarded as waste [1]. Just in 2016, FAO stats showed that 1.45 M tons of pineapple were imported to Europe, and 50% of such amount was used for processing. The standard pineapple processing generates around 60% (*w/w*) of by-products (435,000 tons), estimated in 360 M € economic losses, that were targeted to animal feed, disposed of as waste in landfills, or burned for energy production. Also, in 2016, the European Union launched new waste-management targets towards the transformation of the linear economy into a circular economy. In that sense, by 2030, all the industries have to show that they are applying strategies towards the reuse and recycling of their waste, being at least 30% more efficient than 2014 [2].

Pineapple and its by-products (peels, core, stems, trimming, and crowns) contain several biomolecules of commercial interest, being the enzymes (mainly bromelain) among the most recognized [3]. Stem bromelain (EC 3.4.22.32) is a proteolytic enzyme that belongs to the cysteine group, with several industrial applications. Bromelain is a high-value molecule since it can be applied in different processes in the food industry; for instance, it can be applied for beer clarification, for gluten degradation in baking, for meat tenderization, or improvement of cheese properties. In the animal feed industry, it can be applied for protein hydrolysis and palatability enhancement. Likewise, bromelain finds its place in the pharmaceutical, cosmetic, and nutraceutical industries, given its anti-inflammatory, joint support, and digestive aid properties [4,5].

Other molecules present in pineapple by-products have a relevant impact when strategically applied. Pineapple has a high content of vitamin C, polyphenols, dietary fiber, simple and complex sugars that may be applied for the production of new products [6,7,8,9,10,11,12]. Moreover, the pineapple by-products are constituted by structural molecules, such as insoluble fiber, which can be applied in the production of several foods [13].

Some authors have described the application of pineapple waste as a substrate not only of bromelain but also for ethanol, sugars, vitamins, and growth factors [13]. Moreover, pineapple by-products have been studied as a low-cost substrate for the production of various organic acids, especially citric, lactic, and ferulic acids, using fermentation technology due to the commercial value of these products in the market since they are widely used in food, pharmaceutical, and beverage industries for acidification and enhancement of flavor [13,14].

Pineapple by-products have been described to have a high content of structural carbohydrates—cellulose, hemicellulose, and lignin—which can have different applications. Nowadays, the production of cellulosic materials has highly increased due to the development of renewable and eco-friendly sustainable materials [13]. Thus, the content analysis of cellulose and hemicellulose in new sources, such as industrial fruit by-products, are fundamental for the development of new potential uses through the next years.

There is a considerable gap between the acquired scientific knowledge in this field and the final commercial application. Until now, several works have described the biological and functional properties of fruits [13], especially the pineapple by-products as a possible alternative for new sources of cellulose and hemicellulose [13]. Nevertheless, the new methodologies and technologies applied have high processing costs being not suitable for a smooth implementation on an industrial scale. For the first time, regarding all the legal and economic aspects, the applicable and innovative proposal of pineapple by-products valorization to direct application to the industries was built. The proposal had an easy scale-up, easy application, low cost, and environmentally friendly processes and applications and used a mindset to solve the problems associated with food processing industries.

Therefore, in the present work, the first steps for developing a full and integrated pineapple by-products valorization within a green chemistry approach were given. A full characterization of by-products generated in the industrial environment was applied, followed by the extraction of high-value ingredients, exploiting the potential of the several fractions from industrial processing, allowing a closer approach to a circular economy.

## 2. Materials and Methods

### 2.1. Materials

All the standard polyphenols, standard simple sugars (glucose, mannose, galactose, cellobiose, and fructose), and standard for vitamin C quantification (ascorbic acid, dehydroascorbic acid, cetrimide, potassium dihydrogen phosphates (KH_2_PO_4_), 1,2-phenylenediamine dihydrochloride (OPDA), and orthophthaldehyde (OPA)) were purchased from Sigma–Aldrich (St. Louis MO, USA). The calibration curve of the polysaccharides’ molecular weight was purchased from ShodexTM (Munich, Germany). The 2,2′-azino-bis (3-ethylbenzothiazoline-6-sulphonic acid) (ABTS), 2,2′-azo-bis-(2-metilpropionamidina) dihidrocloro (AAPH), 6-hydroxy-2,5,7,8-tetramethylchroman-2-carboxylic acid (Trolox), Folin–Ciocalteu reagent, fluorescein, and sulfuric acid were purchased from Sigma-Aldrich (St. Louis, MO, USA). The potassium persulfate (K2S2O8) was obtained from Merck (Kenilworth, NJ, USA).

### 2.2. Raw Materials

Fresh pineapple at the ripening stage of ¾ (*Ananas comosus* L.) was purchased from Costa Rica, exported to Portugal, and processed to produced cut-fresh fruit and dehydrate fruit, by a Portuguese national company. In an industrial environment, the pineapple fruit was processed automatically, detaching the crown and stem, and then the skin was mechanically peeled off. The residue parts were frozen at −20 °C for a maximum period of 90 days until further use.

### 2.3. Sample Preparation

**Figure 1:**
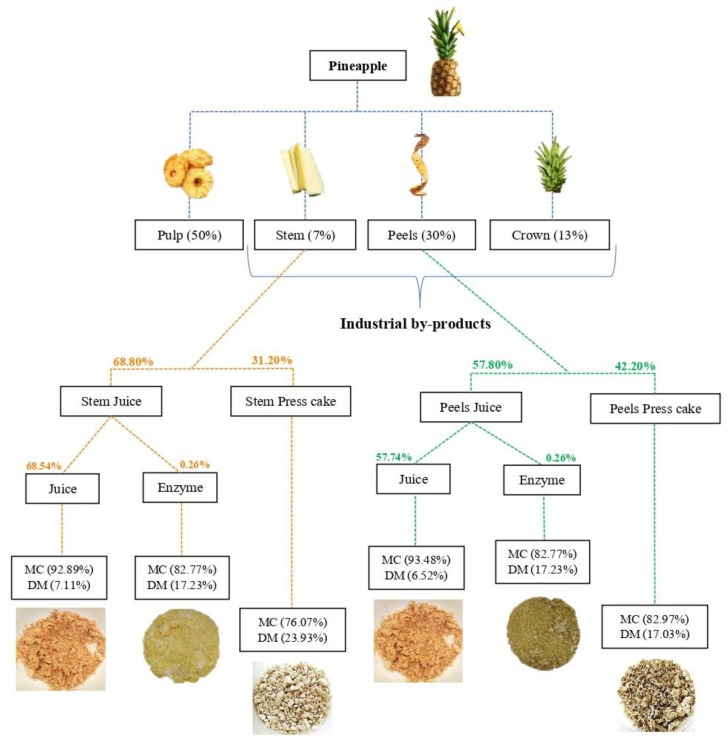
Flow chart of the biomass balance of pineapple by-products (stems and peels) according to an integral valorization towards six functional ingredients. Abbreviations: DM (dry matter) and MC (moisture content).

The pineapple by-product (stems and peels) samples were prepared, as described before by Campos, et al. [15]. Once more, the pineapple by-products were reduced to a juice, using a juice machine (model: MES1020 of 380 W, Bosch, Gellingen, Germany) that separates the solid parts (press cake) from the liquid parts (fruit crude juice), as described in Figure 1. The crude juice was centrifuged, 7370× *g* for 10 min at 4 °C, the obtained supernatant was centrifuged once more for the removal of the pineapple pulp. Solid and liquid fractions were quantified by weighing the mass of three experiments before and after each stage. All fractions were characterized in terms of total simple soluble sugars, pH, and water and protein content, using the analytical procedures given below.

#### 2.3.1. Liquid Phase Separation

Chemical compositional analysis and a complete carbohydrate analysis were performed. An enzyme separation was applied in the liquid fraction, following the methodology described by Campos, et al. [15]. Very quickly, the optimization design of bromelain isolation was studied by the application of electrolyte precipitation, using a natural polysaccharide in pineapple by-products crude juices. In very specific and studied conditions, the efficient precipitation of bromelain was enabled, obtaining high yields of extraction and high enzyme activity recovery [14]. Through the described methodology, it was possible to separate and concentrate the most important enzyme from pineapple—Stem Bromelain. The remaining liquid fraction of both by-products (peels and stems) was evaluated for the antioxidant activity through ABTS and Oxigen Radical Absorbance Capacity (ORAC) assays, as well as the total phenolic content through the Folin–Ciocalteu method. A qualitative and quantitative analysis was performed for the specific polyphenols in the fraction through high-performance liquid chromatography (HPLC). A specific HPLC method was performed to detect and quantify vitamin C derivatives, as described below. An additional analysis through mass fragmentation was performed by LC-ESI-UHR-QqTOF-MS to identify the specific molecules present in pineapple by-products.

#### 2.3.2. Press Cake

The resulting press cake was studied for the structural carbohydrates (Figure 1). An additional evaluation was applied to the press cake to understand the content of simple and complex sugars, as well as the polyphenolic content that remained in the solid fraction. An optimization design was applied to promote hot solubilization in the pineapple press cakes; temperature, time of stirring, and the ratio between press cake and water were studied (data not shown). No significant differences were found between the different extracts (data not shown), and the conditions pursuit were the maximum of the design. Therefore, the insoluble part was submitted to hot aqueous extraction during 2 h at 80 °C with uniform stirring (120 rpm), promoting hot solubilization; the mixture was cooled and centrifuged (12,000 *g* for 20 min) (data not shown in Figure 1). In the supernatant, the antioxidant activity (ABTS and ORAC assays), total phenolic content (Folin–Ciocalteu method), and soluble dietary carbohydrates were also evaluated.

### 2.4. Chemical Composition Analysis

Dry matter, moisture, ashes, protein, dietary fibers, carbohydrates, and pH of pineapple by-products fractions were determined in triplicate according to standard procedures, as described below.

#### 2.4.1. Determination of pH, Dry Weight, and Ashes

A digital pH-meter was used for pH determinations. Dry weight was determined at 105 °C for 24 h; the method was performed according to the Association of Official Analytical Chemists (AOAC) [16]. Moisture content was obtained by the difference between dry and fresh weight. Ash was determined by carbon removal using an initial 2 g of dry matter subject to heating for 5 h in a muffle at 525 °C [16].

#### 2.4.2. Determination of Total Soluble Solids, Total Protein, and Amino Acids

Total sugars were determined by the colorimetric phenol-sulfuric method, as described by Dubois, et al. [17], using glucose (Sigma-Aldrich, St. Louis, MO, USA) as standard. Total nitrogen (N) of the pineapple by-products was measured via the micro-Kjeldahl method. Protein was calculated using a conversion factor (6.25), using a kjeltec system 1002 distilling unit (Tecator; Hogänäs, Sweden). The free amino acid content of each fraction was quantified by pre-column derivatization with orthophthaldehyde (OPA) methodology. Isoindole-type fluorescent derivatives were found in an alkaline solution (borate buffer pH 10.4) from OPA, 2-sulfanylethanol, and the primary amine group of the amino acids. The derivatives were separated by reverse phase-HPLC (Beckman Coulter, Brea, CA, USA) coupled to a fluorescence detector (Waters, Milford, MA, USA), according to the procedure of Proestos, et al. [18]. A hundred microliters of each sample, at concentration 10 mg/mL, were derivatized according to the method, and 20 µL was used as an injection volume of derivatives. All analyses were made in triplicate. The identification and quantification were based on the comparison between retention time and quantified using a calibration curve built with standards of pure amino acids (Sigma-Aldrich, St. Louis, MO, USA).

### 2.5. Determination of Carbohydrates Composition

#### Determination of Dietary Soluble Fiber and Insoluble Fiber

The determination of cellulose, hemicellulose, and lignin was performed in a two-step sequential acid hydrolysis, performed to acquire the structural carbohydrates (sugars). Simple sugars (glucose, xylose, galactose, arabinose, and mannose) were identified and quantified by HPLC analysis. The quantification of cellulose was obtained by the total concentration of glucose, and the hemicellulose was obtained by the total sum of four simple sugars (xylose, galactose, arabinose, and mannose) [19]. On the other hand, the insoluble fiber content was determined to quantify the lignin concentration obtained through gravimetry, after residue filtration. Therefore, the soluble lignin was estimated by UV spectrophotometry at 340 nm, as described before by Sluiter [19]. Moreover, an additional step in two sequential stages was added to the carbohydrate’s evaluation; the water-soluble carbohydrates (simple sugars) and the ethanol-soluble carbohydrates (soluble complex sugars) were removed by using ultrapure water and absolute ethanol [18,19]. These two steps allowed the total quantification of each fraction and the elimination of false positives in the total amount of soluble dietary fiber (SDF).

### 2.6. Assessment of Carbohydrates by HPLC

#### Determination of Simple Sugars

The chromatographic analysis was performed using a Beckman and Coulter 168 series HPLC system interfaced with a photodiode array UV/Vis detector (PDA 190–600 nm) (Beckman and Coulter; Fullerton, Brea, CA, USA). The separation was done using an Aminex HPX-87H column (BioRad, Hercules, CA, USA), with H_2_SO_4_ as a mobile phase at 0.003 mol/L, with a flow of 0.6 mL/min. An oven was applied to the column at 60 °C, allowing better separation of monosaccharides. Data acquisition and analysis were accomplished using Karat32 software. The detection was performed with an infrared detector (Knauer, Berlin, Germany); peaks were analyzed and quantified using calibration curves of each monosaccharide. The peaks obtained were analyzed by comparison of retention time with standards of simple sugars (glucose, fructose, mannose, galactose, cellobiose, arabinose, and xylose). Three independent analyses were performed for each experiment.

### 2.7. Determination of the Antioxidant Capacity

#### 2.7.1. Fluorometric Method (ORAC Method)

The antioxidant activity of pineapple by-products liquid fraction and hot aqueous extraction from press cake were measured by ORAC assay, according to Dávalos, et al. [20]. This method is based on the oxidation of fluorescein by peroxide radicals produced in situ by thermal decomposition of AAPH. Fluorescein solution was prepared at a concentration of 116.66 mM from a stock solution of fluorescein 1166.1 µM in 75 mM PBS (pH 7.4). The antioxidant acid-Trolox was prepared in 0.1 mM PBS and used as a positive control. This solution was diluted to obtain serial concentrations (0.0002, 0.0004, 0.0006, 0.0008, 0.0010, 0.0012, 0.0014, and 0.0016 µmol) for the calibration curve. The AAPH was dissolved in 0.1 M (pH 7.4) to a final concentration of 46.6 mM. A total of 20 µL of sample or buffer (in the case of the control) was mixed with 120 µL of fluorescein and 60 µL of AAPH in a microplate black polystyrene (Nunc, Roskilde, Denmark) and incubated at 40 °C. During 137 min, 104 measurements of fluorescence were taken in a Fluostar Optima fluorimeter (BMG Labtech, Offenburg, Germany) at an excitation wavelength of 485 nm and 520 nm of emission. The software used was the Fluostar Control version 1:32 R2. All samples were run in triplicate for each experiment.

#### 2.7.2. ABTS Assay

The total antioxidant activity of pineapple by-products liquid fractions, as well as the hot aqueous extract of press cake, was measured by the *ABTS* radical cation decolorization assay, as described by Re, et al. [21]. *ABTS* was dissolved in water at a final concentration of 7 mM. ABTS radical cation (*ABTS* •+) was produced by reacting *ABTS* stock solution with 2.45 mM potassium persulfate (Merck, Darmstadt, Germany) (final concentration) and kept in the dark at room temperature (25 ± 2 °C) for 12–16 h before use. The radical maintained a stable form for more than two days when stored in the dark. Before analysis, (*ABTS* •+) was filtered using a 0.22 µm filter (Orange Scientific, Braine-l’Alleud, Belgium) and diluted with redistilled water to an absorbance of 0.700 ± 0.02 to 10 µL of the sample, and the absorbance was read exactly 6 min after initial mixture. Since the inhibition percentage (*IP*) must be between 20% and 80%, the samples were diluted when needed. These values were then calculated using a calibration curve prepared using standard solutions of ascorbic acid. All assays were performed in triplicate for each experiment.

(1) $$IP=\frac{OD\;\left(diluted\;ABTS•+\;\right)\;-OD\;sample}{OD\;\left(diluted\;ABTS•+\;\right)}\;\times \;100,$$

### 2.8. Quantification of Total Polyphenol

#### 2.8.1. Folin–Ciocalteu Method

The total polyphenol content of the pineapple by-products liquid fraction and hot aqueous extract was measured using a modified Folin–Ciocalteu colorimetric method described by Gao, et al. [22]. Aliquots of 50 µL of samples and control (distilled water) were mixed with 50 µL of Folin–Ciocalteu reagent (0.25 N), 1 mL of sodium carbonate (1N), and 1.4 mL of distilled water, all added in this exact order. The total polyphenol content was determined after 1 h of incubation at room temperature (25 °C). The absorbance of the resulting blue color was measured at 750 nm by colorimetry using an UVmini 1240 UV-Vis spectrophotometer (Shimadzu, Washington, DC, USA). A standard curve was performed using different concentrations of gallic acid. All measurements were performed in triplicate for each experiment.

#### 2.8.2. Vitamin C Determination

Ascorbic acid (AA) and dehydroascorbic acid (DHAA) contents on pineapple by-product liquid fraction and hot aqueous extract were assessed through HPLC-DAD (diode array detector) (Water Series 600, Amherst, MA, USA), as described by Zapata and DUFOUR [23]. The total determination of vitamin C content (AA+DHAA) was achieved after the derivatization of DHAA into the fluorophore 3-(1,2-dihydroxy ethyl) furol [3,4-b] quinoxaline-1-one (DFQ), with 1,2-phenylenediamine dihydrochloride (OPDA). The separation was done in a C18 reverse-phase column coupled with a guard column (pore size 100 Å, particle size 5 µm, lengths 4.6 mm × 150 mm), containing the same stationary phase (Symmetry^®^ C18, Waters, Milford, MA, USA). The mobile phase was a mixture of water and methanol (95:5, % *v/v*), containing 5 mM cetrimide and 50 mM potassium dihydrogen phosphate, at pH 4.5 at a flow rate of 1 mL/min. The detection for AA was performed at 261 nm and for DHAA at 348 nm. The identification and quantification were performed by comparison with standards and calibration curves. All measurements were performed in triplicate for each experiment.

#### 2.8.3. Phenolic Compounds Identification by HPLC

The phenolic profile of the pineapple by-products (peels and stems) liquid fraction were evaluated using a Waters e2695 separations module system interfaced with photodiode array UV/Vis detector (PDA 190–600 nm), according to the method described previously by Campos, et al. [24], and changes in the original method were applied to better separate the pineapple phenolic compounds.

The separation was done in a C18 reverse-phase column coupled with a guard column (pore size 100 Å, particle size 5 µm, lengths 4.6 mm × 150 mm), containing the same stationary phase (Symmetry^®^ C18, Waters, Milford, MA, USA). Chromatographic separation of phenolic compounds was carried out with mobile phase A-water, methanol (Panreac, Barcelona, Spain), and formic acid (Merck, Darmstadt, Germany) (92.5:5:2.5; % *v/v*) and mobile phase B-methanol, water, and formic acid (92.5:5:2.5; % *v/v*). The runs were made by the following conditions: gradient elution starts at 100% mobile phase A and ends at 55% mobile phase B after 55 min at a continuous flow 0.5 mL/min, between 50 and 55 min the mobile phase A returns to 100% and remains at this percentage for 4 min (until 59 min). The injection volume was 20 µL. Detection was achieved using a diode array detector (Waters, Milford, MA, USA) at wavelengths ranging from 200 to 600 nm measured in 2 nm intervals. The peaks were searched for several wavelengths to identify catechins or procyanidins (280 nm), phenolic acids (320 nm), flavonols (330 nm), and anthocyanins (520 nm) and were analyzed by comparison of retention time and spectra with pure standards. Three independent analyses were performed for each experiment.

### 2.9. Identification of Phenolic Compounds by Mass Fragmentation

Phenolic compounds also were identified by LC-ESI-UHR-QqTOF-MS in the pineapple by-products liquid fraction, according to Monforte, et al. [25]. For the characterization of the samples, an UltiMate 300 Dionex UPLC (Thermo Scientific, Waltham, MA, USA), coupled to an ultra-high resolution Qq-time-of-flight (UHR-QqTOF) mass spectrometer with 50,000 full-sensitivity Resolution (FSR) (Impact II, Bruker Daltonics, Bremen, Germany), was used. The identification of the molecules was performed using an Acclaim RSLC 120 C18 column (100 mm × 2.1 mm, 2.2 µm) (Dionex). The injection volume was 1 µL. Two mobile phases were employed—the mobile phase A consisting of water and formic acid (99.9:0.1; % *v/v*), and mobile phase B of acetonitrile and formic acid (99.9:0.1; % *v/v*). The gradient started in 5% of mobile phase B until 95% during 7 min, which was maintained constant for 2 min and returned to 5% mobile phase B in 1 min and maintained at 5% for 5 min more at a flow rate of 0.25 mL/min. The parameters were set, as described by Monforte, et al. [25], using positive ionization mode with spectra acquired over a range from *m/z* 20 to 1000. A 4.5 kV of capillary voltage was applied, and the temperature was 200 °C in the drying gas, drying gas flow was 8 L/min, the pressure was 2 bar in the nebulizing gas, 300 Vpp in collision RF, with a 120 µs of transfer time and pre-pulse storage of 4 µs. A syringe pump delivered post-acquisition internal mass calibration using sodium formate clusters with the sodium formate at the start of each chromatographic analysis. Also, high-resolution mass spectrometry (MS) was used to identify the phenolic compounds present in the fractions. The elemental composition for the compounds was confirmed according to accurate mass and isotope rate calculations designated as mSigma (Bruker Daltonics, Billerica, MA, USA). The accurate mass measurement was within 5 mDa of the assigned elemental composition, and mSigma values of <20 provided confirmation. Identification was carried out using pure standards (1 mg/L) in methanol (LC-MS grade). All the phenolic compounds were identified on their accurate mass [M − H]^−^.

### 2.10. Statistical Analysis

The experiment results were analyzed through statistical analysis performed by SPSS 23.0 software (IBM, Armonk, NY, USA). The normality of data distribution was tested by the Shapiro–Wilk test, and statistical significance values were analyzed using a Student’s *t*-test, considering a significant level of 5%.

## 3. Results and Discussion

### 3.1. Mass Balance and the Physicochemical Composition of Pineapple By-Products

Pineapple processing generates every day tons of waste, represented mainly by the crown (leaves), peels, and stems. In this experimental work, pineapple peels (ca. 30%; *w/w*) and stems (ca. 7%; *w/w*) were characterized. The crown was not evaluated because it was removed in the production field. Figure 1 shows the by-products’ fractions and the mass percentage of each fraction, as well as the production of six new ingredients from the pineapple by-products (three for each by-product). The physicochemical composition of peels and stems was evaluated, and it is presented in Table 1. The fruit variety and maturity stage could influence these values, as described by Lombardi-Boccia, et al. [26] who found differences on total mineral content between the same fruits and by-products because of the type of soil used for cultivation. Therefore, some differences in the physicochemical composition were expected when compared to the actual results with the bibliography.

The moisture content of each by-product was evaluated, being in line with values previously reported by Huang, et al. [27] for fresh pineapple peels. Concerning the chemical composition of both by-products, slight differences were found for proteins and carbohydrates, with range values similar to those described by Huang, et al. [27] in the compositional analysis of pineapple peels for total carbohydrate and total protein contents.

The pineapple by-products were applied to a juice machine for liquid extraction, generating two press cakes (peels and stems) and two liquid fractions (peels and stems). The liquid fractions were submitted to precipitation with natural polysaccharides for enzymatic extraction, as described before by Campos, et al. [15]. The most important enzyme of pineapple is stem bromelain, which is present mainly in stem and core [28]. Through the precipitation, it was possible to extract mainly stem bromelain and minor amounts of fruit bromelain, with high transfer yields from the total juice to the precipitate [15]. The enzymatic fraction represented 0.26% (*w/w*) of the whole pineapple.

**Figure 2:**
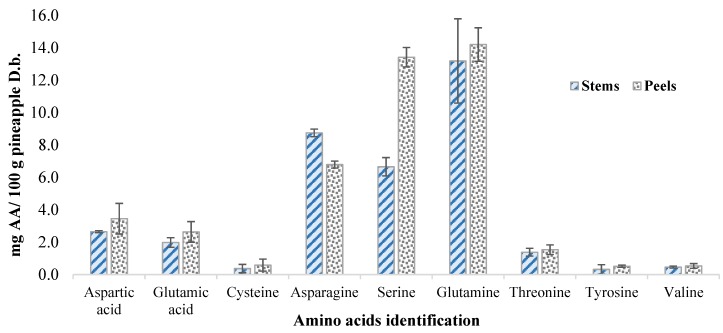
Identification and quantification of free amino acids of pineapple by-products (stems and peels). Abbreviations: D.b—Dry basis; AA—Amino Acids.

After enzymatic precipitation, the remaining juice still presented the high content of bioactive molecules. Therefore, an integrated valorization with a functional approach was searched. The remaining liquid fractions of stem and peel were ca. 68.54% (*w/w*) and ca. 57.74% (*w/w*), respectively, which represented in weight ca. 4.80% (*w/w*) and ca. 17.26% (*w/w*) of the total pineapple. Thus, the total amount of the liquid extracted from total pineapple by-products was ca. 22.06% (*w/w*) (Figure 1). The remaining liquid fraction was evaluated and characterized for total carbohydrates (simple sugars and soluble dietary fiber) (Table 2), polyphenols, antioxidant capacity (Table 4), and the amino acid profile (Figure 2).

### 3.2. Solid Fraction Evaluation

The press cake that resulted from the juice extraction of both by-products was also evaluated and characterized, mainly for the chemical composition regarding the structural carbohydrates. After juice extraction, the structural fibers (Table 2 and Table 3) mainly composed the remains.

Then, the press cakes were evaluated for the total content of structural carbohydrates (Table 3), and differences in the cellulose content were found. Thus, the stem presented significant (*p* < 0.05) higher content of cellulose than peels. Other authors have reported the percentage of cellulose in pineapple by-products, showing similar values. Larrauri, et al. [29] reported ca. 23% (*w/w*) of cellulose in the pineapple peels, while Huang, et al. [27] reported ca. 18% (*w/w*). Further, both fractions presented similar values in the total dietary fiber. Statistically, differences (*p* < 0.05) were found in the content of insoluble dietary fiber (cellulose and hemicellulose). The stem press cake presented ca. 44% (*w/w*), and peel press cake ca. 38% (*w/w*). Furthermore, the content of soluble lignin was similar between peels and stems (*p* > 0.05).

### 3.3. Liquid Fraction Evaluation

The total carbohydrates were evaluated for the liquid fraction after protein extraction. Results showed that the simple sugars concentration increased ca. 65% (*w/w*) in the stem liquid fraction and ca. 67% (*w/w*) in the peel liquid fraction, while the soluble dietary fiber decreased when compared with the solid fraction (press cake) (Table 2). This is an expected result because the final step of protein separation is centrifugation; therefore, a separation between supernatant from the precipitate occurs. The soluble dietary fiber is associated with the pulp on the fruit; consequently, the pulp in suspension joins the protein fraction in the precipitate.

### 3.4. Determination of Free Amino Acids

Quantitative and qualitative determination of free amino acids was performed by HPLC (Figure 4). Amino acids affect the quality of foods, but also are useful markers to define fruit juice genuineness [30]. Eleven free amino acids were identified, and nine were quantified (it was not possible to quantify histidine and glycine). The total amount of free amino acids for pineapple peel liquid fraction was ca. 46.7 mg a.a./100 g dry basis, and for the stem liquid fraction, it was 38.5 mg a.a./100 g dry basis. Dizy, et al. [31] reported the number of free amino acids in fruit juices, with a higher concentration of free amino acids for commercial pineapple juices. The amino acids with higher concentrations present in the juices were asparagine, serine, and aspartic and glutamic acid. In this work, the most impacting amino acids were also asparagine, serine, and aspartic and glutamic acid, but also glutamine, conforming with Dizy, et al. [31] and Fabiani, et al. [30]. The main difference between fractions was found only for the concentration of serine, where peels liquid fraction presented higher concentration, as can be seen in Figure 2.

### 3.5. Determination of Free Total Phenolics Compounds and Antioxidant Capacity

The liquid fractions were also characterized for total phenolic compounds by Folin–Ciocalteu method, and the antioxidant activity was evaluated by ABTS and ORAC assays (Table 4). The pineapple peel liquid fraction presented a lower amount of total phenolics when compared with stem liquid fraction, which demonstrated to have the double amount of total phenolic compounds. Other authors also have reported the total amount of polyphenols in pineapple by-products; Almeida, et al. [32] reported 298.6 mg Gallic acid equivalents (GAE)/100 g dry basis, which is lower than the values obtained in this work, while da Silva, et al. [33] reported 2784.1 mg/100 g dry basis, which is very different from the ones reported in Table 4. Differences between works were due to the methanolic extraction, which is usually applied to achieve the highest yields of extraction of phenolic compounds from natural samples, which was the case of Almeida, et al. [32] and da Silva, et al. [33]. Nevertheless, Almeida, et al. [32] presented lower values of temperature to do the methanolic extraction, as described by Georgé, et al. [34], who reported that the application of high temperature (above 80 °C) led to elimination of vitamin C, obtaining more accurate results for total phenolic compounds. As described previously in the section of Materials and Methods, to the solid fractions (press cake), a hot water extraction (80 °C, during 2 h) was applied, and the remaining liquid was evaluated for antioxidant potential. Results showed a decrease of total phenolic compounds and the disappearance of vitamin C, along with a decrease of antioxidant capacity (Table 4), which was in line with Georgé, et al. [34], who reported that high temperature led to decrease of total phenolic content and elimination of ascorbic acid derivatives.

The same tendency was noticed for antioxidant capacity by ABTS assay when comparing liquid fraction with hot aqueous extracts, and the stem liquid fraction presented four times higher antioxidant activity than peel liquid fraction. In the evaluation of antioxidant capacity through ORAC assay, differences were not found between both liquid pineapple fractions. This discrepancy between the presented results of ABTS and ORAC, where ABTS presents lower values of equivalents, was expected since both methods present different reaction mechanisms [35]. ABTS radical presents a higher molecular weight when compared with the molecule of transfer mechanisms of hydrogen-atom of ORAC; therefore, a steric block of the active centers of ABTS can occur, decreasing the rate of reaction of this assay. Therefore, due to the small molecules of atom transfer of ORAC assay, this method has higher accuracy in the antioxidant capacity, as previously described by Campos, et al. [24] and Coscueta, et al. [35].

The antioxidant capacity of fruits and by-products may change, depending on their content of vitamin C, vitamin E, carotenoids, flavonoids, and other polyphenols [36]. Therefore, pineapple by-products hot water extracts and liquid fractions were evaluated for vitamin C content through HPLC analysis. The vitamin C was calculated through the quantification of AA and DHAA. Ascorbic acid derivatives were not detected for hot aqueous extraction of a press cake of pineapple by-products. Moreover, the stem liquid fraction showed a higher concentration of total vitamin C than peel liquid fraction. Nonetheless, the proportion of AA/DHAA was different between by-products, with stem liquid fraction, presenting a higher concentration of AA, while peel liquid fraction, presenting a higher concentration of DHAA.

### 3.6. Determination of Free Polyphenols

The liquid fraction of stems and peels were characterized for polyphenols by HPLC analysis, using an adapted method specific for pineapple juices, as described above. The evaluation was performed directly in the liquid fractions without any extraction. Phenolic compounds were identified by comparison of UV spectra and retention times with those of known standards. Quantification was made, when it was possible, according to the area method at the wavelength, where the highest response was obtained. Moreover, some phenolics in the pineapple by-products’ liquid fractions were identified and not quantified (below quantification limit, BQL), as described in Table 5. Moreover, several wavelengths for total identification in polyphenols ranges were searched, as described previously, but the peaks were detected only at 280 and 320 nm, an expected result due to the water-based pineapple samples (hydrophilic molecules).

**Figure 3:**
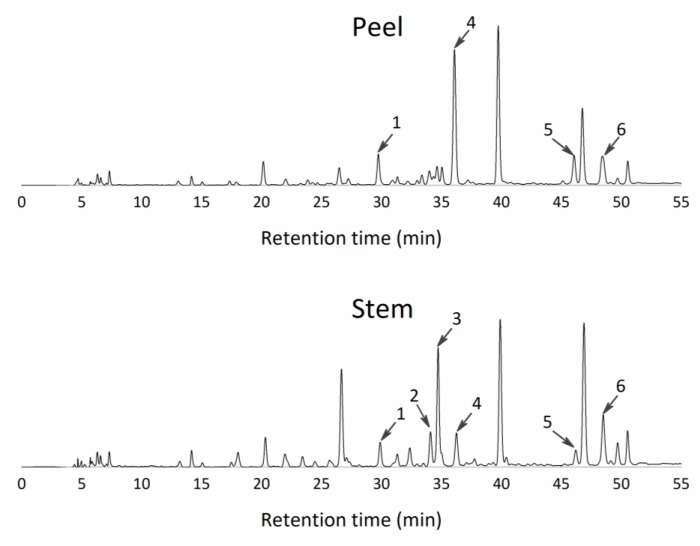
Chromatograms of the main peaks found and identified as free phenolic compounds of pineapple by-products at a wavelength of 320 nm. The liquid fraction of peels and the liquid fraction of stems. 1—Chlorogenic acid; 2—p-hydroxybenzoic acid; 3—2,5-dihydroxybenzoic; 4—Caffeic acid; 5—Syringaldehyde; 6—Ferulic acid.

**Figure 4:**
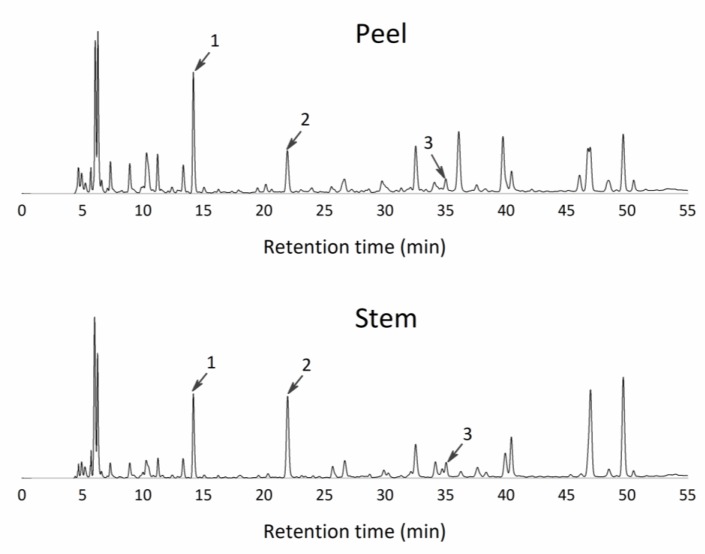
Chromatograms of the main peaks found and identified as free phenolic compounds of pineapple by-products at a wavelength of 280 nm. The liquid fraction of peels and the liquid fraction of stems. 1—Gallic acid; 2—Hydroxytyrosol; 3—2,5-dihydroxybenzoic.

In the pineapple stem liquid fraction, eight polyphenols were identified at the 320 nm wavelength (Figure 3), of which only two compounds could be quantified—p-hydroxybenzoic acid and ferulic acid (Table 5). At the 280 nm wavelength, two peaks were identified but not quantified—gallic acid and hydroxytyrosol (Figure 4). On the other hand, in the pineapple peel juice, seven peaks were detected and identified (Figure 3). In the 320 nm analysis, five peaks were found, but only two were quantified—caffeic and ferulic acid (Table 5). Therefore, the main polyphenols found in the pineapple by-products juices (water-based samples) were from the hydroxybenzoic acid group and the hydroxycinnamic acid group.

Other authors have reported the polyphenol profile in pineapple by-products. Li, et al. [37] identified and quantified four peaks in methanolic extracts of pineapple peels—gallic acid, catechin, epicatechin, and ferulic acid. Two of those peaks were in line with the ones found in this work, but the remaining peaks (flavanols) were not found because catechins are extracted more efficiently using organic solvents, such as methanol. Moreover, Hossain and Rahman [38] described the different amounts of polyphenols extracted from pineapple peel with different solvents; methanol was the best solvent, and water was the worst since presented a yield of extraction of ca. 4.3% (*w/w*). Also, Sopie, et al. [39] characterized different parts of pineapple by HPLC analysis, using methanol to extract polyphenols. The authors identified eight phenolic compounds—gallic, gentisic, syringic, vanillic, ferulic, sinapic, isoferulic, and o-coumaric acids—all derivative compounds from hydroxycinnamic and hydroxybenzoic acids.

Differences in polyphenol profile between this work and other previous works were expected because the different applied extraction methodologies, the concentration of the tested samples, and the environmental factors affect the polyphenolic profile of pineapple fruit and by-products. Moreover, several works described the presence of polyphenols bounded with dietary fiber. Larrauri, Rupérez, and Calixto [29] studied differences between free and bounded polyphenols in pineapple by-products, showing that a high percentage of polyphenols were bounded with the dietary fiber from pineapple, and the total content of polyphenols was higher, as well as the antioxidant capacity of the tested samples.

### 3.7. Determination of Mass Fragments

Several peaks in the HPLC analysis were not identified using standard compounds typical from pineapple and other fruits. Therefore, a more in-depth analysis was performed to identify more polyphenols pineapple by-products. Larrauri, et al. [29] described the presence of bounded polyphenols with dietary fiber, which also means that several polyphenols could be glycosylated to become more stable. This kind of structural modification leads to a difference in the HPLC patterns (retention time and different UV maximum absorbance), being difficult the identification of the compounds. The mass spectrometry instrument was used to provide the exact mass precursor and fragment ion information, allowing elucidation of potential structures of unknown pineapple metabolites. The accurate mass information of precursor and product ions was used to calculate the proposed molecular formula of each compound. Therefore, the same samples were evaluated through LC-MS, and the results are summarized in Table 6. All suggested formulas had excellent mass accuracy, less than 2 mDa (data not shown), increasing the confidence of the predicted compounds (Table 6). Nineteen molecules were identified and grouped by amino acids, phenolic compounds, non-phenolic compounds, and other molecules.

#### 3.7.1. Amino Acids

L-Tyrosine (no.1) and L-Tryptophan (no.6) were identified through MSn spectrometry. The presence of L-Tryptophan was corroborated by Steingass, et al. [41], while the presence of both amino acids was corroborated by Jandrić, et al. [42] as usual amino acids presented on pineapple.

#### 3.7.2. Phenolic Compounds

Phenolic compounds of pineapple by-product liquid fractions were identified through mass fragmentation, crossing accurate mass and UV maximum absorbance. Three compounds were quantified by UPLC analysis in both pineapple by-products: caffeic acid (no. 8), coumaric acid (no. 15), and ferulic acid (no. 18) (concentration described in Table 6), in line with the results obtained previously in the HPLC analysis. Synapoyl hexoside (no. 10), 2-caffeoyl isocitrate (no. 12), S-coniferyl glutathione (no. 16), and N-L-γ-glutamyl-S-synapyl-L-cysteine (no. 17) are polyphenols and were identified by cross-linking our database. These four molecules are typically recognized as pineapple polyphenols.

The analysis suggested that most compounds, caffeic, ferulic, synaptic acid derivatives, as well as compounds no. 10 and no. 12, were hydroxycinnamoyl glycosides. The same molecules were identified by Steingass, et al. [40] and lately confirmed by Jandrić, et al. [42] and Difonzo, et al. [43]. The caffeoyl aldarate (no. 3) and feruloyl aldarate (no. 7) were identified, also as hydroxycinnamoyl glycosides, but it was not previously reported as pineapple constituents. On the other hand, the glutathione derivatives and the L-cysteine derivatives have been identified by other authors as typical pineapple polyphenols; in this work, they were identified as the compound no. 16 and no. 17 [40,43].

#### 3.7.3. Other Compounds

Also, a non-phenolic compound was identified—1-(1H-Pyrrole-2-carboxyl)-glucuronosyl glycerol—with a mass profile that matches with that reported by Steingass, Jutzi, Müller, Carle, and Schmarr [40]. Likewise, compound no. 14, with [M − H]^−^ precursor ions at *m/z* 371, 249, 175, and 121, was not identified, and the result was also corroborated by the research work of Steingass, Jutzi, Müller, Carle, and Schmarr [40] and Difonzo, Vollmer, Caponio, Pasqualone, Carle, and Steingass [43].

One lipid was identified—6-O-(2-Hydroxyhexanoyl)-D-glucopyranose (no.4)—with [M − H]^−^ precursor ions at *m/z* 293, 251, 119, and 101. Considering the database of LC-MS, the identified lipid was a fatty acyl glycoside of mono and disaccharide, having the common name ethyl 3-O-β-D-glucopyranosyl-butanoate. This lipid was identified for the first time in pineapple peel juice.

Other phenolic compounds, unknown to our database, were observed. Considering the database of LC-MS, their possible structure and identity were proposed. The [M − H]^−^ precursor ions at *m/z* 315, 248, 153 was proposed as 3,4-Dimethoxyphenyl β-D-glucoside, and *m/z* 243, 199, 130, with UV maximum absorbance of 270 nm and retention time of 7.8 min and *m/z* 243, 199, 157, 130, with 270 nm at retention time were proposed as 6-[(6-Aminohexanoyl) amino] hexanoate. The [M − H]^−^ precursor ions at *m/z* 376, 228, 211, and 164, with UV maximum of 317 nm and retention time of 12.4 min was proposed as N-[(Benzyloxy)carbonyl] leucyileucin amide. Eugenin was identified at the stem liquid fraction, and it is a well-known polyphenol recognized from plants as one of the main responsible for bitterness.

Some differences were expected in the mass fragmentation between the pineapple by-products (peel and stem), but also some similarities. Pineapple stem is the connection between the pineapple crown with the fruit, while the transition between pineapple peel and pulp are not clearly defined; therefore, compounds only identified in the pulp or stem parts may also be detected in the outer parts [43].

### 3.8. Value-Add of the Pineapple By-Products Ingredients

Several fractions were developed from pineapple by-products studied. The fractions were characterized, and three natural ingredients were developed from each by-product, creating a total of six ingredients, as can be seen in Figure 1. More than this, the applied approach enabled the 100% valorization of pineapple by-products, reducing the negative environmental impact of deposition of such material in landfills.

The enzymatic fraction obtained through polysaccharide precipitation, as described by Campos, et al. 2019, enabled the creation of high purity grade stem bromelain extract, with high enzymatic activity. Bromelain is a special and well-known enzyme in the global market, and it is anticipated to reach 945 million euros by 2025, with a demand of 579,958.3 Kg [44] since is applied in food, nutraceutical, pharmaceutical, chemical, and textile industries. Nowadays, it is mostly obtained by extraction from all fruit.

The juices were a result of bromelain extraction, and the by-product of such extraction was a juice that remained exactly the same as the original with the absence of enzyme. Once dried, the functional properties of this fraction were studied for the application as a value-add ingredient for industrial use in food and nutraceutical. The pineapple by-product juices presented a high concentration of phenolic compounds with high antioxidant activity and high content of vitamin C (Table 4), and the great concentration of soluble dietary fiber was added to this that also is well known for the beneficial effects associated with human health. The dried juice has a high potential application for the enhancement of flavor in foods and formulations for nutraceutical industries. Also, the high content of soluble dietary fibers in fruits and by-products have been studied due to the potential benefits of dietary fibers, and a wide range of fiber-rich products have been developed as functional foods. The dietary fiber is associated with colorants, antioxidants, and other positive effects molecules that can have a synergetic health effect [45,46].

The flours were a result of the wet pulp (press cake) that was dried and ground, producing fine powders that could be applied for technological and functional improvements of foods in food industries as a natural ingredient, or as a substitute of cereal flours. On the other hand, the search for natural ingredients by the consumers has increased, and fruit by-products’ flours can be an excellent substitute for this new market niche. The press cake of pineapple by-products showed high content of IDF, as can be seen in Table 2. The food industry has searched for new sources of natural IDF as an ingredient to increase the indigestible and insoluble compounds in food products [47]. From a human consumption point of view, the consumption of foods with a high amount of IDF increases the satiety and volume of foods, as well as promotes the functioning of the digestive system [45]. The flours produced from the press cake present a viable option for food industries for the production of reduced calories and dietary fiber-enriched meals [27,47].

## 4. Conclusions

Pineapple waste is rich in bioactive molecules; thus, the conversion of such materials into wealth is the ongoing interest of the scientific field.

The physicochemical analysis provides a more in-depth characterization of pineapple by-products (peel and stem), making possible the development of a sustainable process for fruit by-product valorization through fractionation.

The application of an adequate separation operation to separate the liquid and solid is crucial to develop functional fractions. The liquid fraction, which represents ca. 57.8% (*v/w*) of peel and ca. 68.8% (*v/w*) of the stem, can be divided into two ingredients—the enzymatic fraction and the remaining liquid. The first offers a stem bromelain concentrate, while the second an excellent source of polyphenols and soluble dietary fiber that can be applied as a prebiotic enhancer and antioxidant agent.

The solid fractions from pineapple peel and stem have excellent potential in food applications as a functional ingredient, especially in the development of food reduced in calories and dietary fiber-enriched food products.

In summary, pineapple by-product fractions are a promising source of valuable bioactive compounds. Dietary fiber is an interesting target to valorize, but also the insoluble fiber-containing associated polyphenols that exhibit antioxidant activity. These properties together, as well as the neutral color and flavor of pineapple, make it a suitable source of several bioactive and functional ingredients for a wide range of applications, either for the food industry or for cosmetics and nutraceutical industries.

## Figures and Tables

**Table 1:** Physicochemical composition (average ± standard deviation) of pineapple by-products (peels and stems) in fresh fruit. Results were given in dry basis.

Pineapple By-Products	Stem (% *w/w*)	Peel (% *w/w*)
Insoluble fibers	6.54 ± 0.29	5.17 ± 0.26
Carbohydrates	17.16 ± 0.33	13.07 ± 0.44
Soluble dietary fibers	1.10 ± 0.20	1.41 ± 0.35
*Insoluble dietary fibers*	10.64 ± 0.13	6.76 ± 0.10
*Simple sugars*	5.44 ± 0.40	4.90 ± 0.18
Protein	0.98 ± 0.05	0.84 ± 0.02
Fat	n.d.	n.d.
Ash	1.15 ± 0.23	0.99 ± 0.17
Moisture content	76.07 ± 4.93	82.35 ± 0.36
pH	3.65 ± 0.05	3.63 ± 0.02

Abb.: non-detected, n.d.

**Table 2:** Total carbohydrate (average ± standard deviation) composition of pineapple by-products liquid and a solid fraction (peels and stems) in dry basis.

**Solid Fraction (Press Cake)**	**Carbohydrates (% *w/w*)**			**Insoluble Fiber (% *w/w*)**
	**SS**	**SDF**	**IDF**	
Stems	22.68 ^b^ ± 1.68	4.57 ^b^ ± 0.84	44.37 ^a^ ± 0.56	27.28 ^a^ ± 1.19
Peels	27.74 ^a^ ± 1.03	7.99 ^a^ ± 2.00	38.30 ^b^ ± 0.54	29.27 ^a^ ± 1.45
**Liquid Fraction (Crude Juice)**	**Carbohydrates (% *w/w*)**			**Insoluble Fiber (% *w/w*)**
	**SS**	**SDF**	**IDF**	
Stems	65.08 ^a^ ± 1.81	0.45 ^b^ ± 0.04	n.d.	n.d.
Peels	67.15 ^a^ ± 1.52	0.53 ^b^ ± 0.03	n.d.	n.d.

Abb.: Simple sugars, SS; Soluble dietary fiber, SDF; Insoluble dietary fiber, IDF; non-define, n.d.; ^a^, ^b^ The differences between the means in the same column labeled with different superscripts are statistically significant (*p* > 0.05). Analysis of variance was used to estimate the differences between samples. Shapiro–Wilk test was used. Values were expressed as average ± standard deviation.

**Table 3:** Structural carbohydrates composition of pineapple by-products (peels and stems) of the solid fraction (press cake).

Solid Fraction (Press Cake)	Cellulose (% *w/w*)	Hemicellulose (% *w/w*)					Lignin (% *w/w*)	
	Glucose	Total	Xylose	Galactose	Arabinose	Mannose	Insoluble	Soluble
**Stems**	23.88 ^a^ ± 0.22	20.68 ± 0.34	11.05 ^b^ ± 0.22	3.24 ^a^ ± 0.05	3.67 ^a^ ± 0.10	2.72 ^a^ ± 0.02	24.25 ^b^ ± 1.31	3.03 ^a^ ± 0.11
**Peels**	17.41 ^b^ ± 1.73	19.96 ± 0.38	13.80 ^a^ ± 0.19	2.76 ^b^ ± 0.04	2.53 ^b^ ± 0.38	0.91 ^b^ ± 0.14	25.66 ^a^ ± 1.20	3.61 ^a^ ± 0.26

^a^, ^b^ The differences between the means in the same column labeled with different superscripts are statistically significant (*p* > 0.05). Analysis of variance was used to estimate the differences between samples. Shapiro–Wilk test was used. Values were expressed as average ± standard deviation.

**Table 4:** Evaluation of antioxidant capacity (ABTS and ORAC assay), total phenolic compounds (Folin–Ciocalteu method), and total vitamin C (HPLC method) of pineapple by-products crude liquid fraction (stems and peels). All results expressed in mg/100 g on a dry basis.

Pineapple By-Products Fractions		ABTS Assay (mg AAE/100 g)	ORAC Assay (g TE/100 g)	Folin–Ciocalteu (mg GAE/100 g)	Ascorbic Acid (mg/100 g)		Total Vitamin C (mg AAE/100 g)
					AA	DHAA	
Solid fraction (Hot aqueous extraction)	**Stems**	93.4 ± 1.5	2.8 ± 0.1	157.8 ± 4.7	n.d.	n.d.	n.d.
	**Peels**	209.6 ± 4.7	8.1 ± 0.3	302.1 ± 7.6	n.d.	n.d.	n.d.
Liquid fraction (Crude juice)	**Stems**	1290.3 ± 15.9	13.5 ± 0.6	1270.1 ± 11.9	188.9 ± 0	24.0 ± 2.9	121.2
	**Peels**	300.7 ± 22.9	12.5 ± 0.3	652.8 ± 39.9	29.1 ± 0	92.0 ± 13.8	212.9

Abb.: Non-detected, n.d.; Ascorbic acid, AA; Dehydroascorbic acid, DHAA; Ascorbic acid equivalent, AAE; Trolox equivalent, TE; Gallic acid equivalent, GAE.

**Table 5:** Identification and quantification of phenolic compounds present in pineapple by-products by HPLC analysis. Evaluation of stem and peel liquid fractions.

Phenolic Compounds	Chemical Formula	*t*_R_ (min)	λ_max_ (nm)	Stem Juice (mg/L)	Peel Juice (mg/L)
Gallic acid	C_7_H_6_O_5_	14.38	280.0	BQL	BQL
Hydroxytyrosol	C_8_H_10_O_3_	21.41	280.0	n.d.	BQL
Chlorogenic acid	C_16_H_18_O_9_	30.08	325.7	1.31 ± 0.04	0.85 ± 0.07
p-hydroxybenzoic acid	C_7_H_6_O_3_	33.86	329.3	0.82 ± 0.04	n.d.
Cryptochlorogenic acid	C_16_H_18_O_9_	34.69	303.3	BQL	n.d.
2,5-dihydroxybenzoic acid	C_7_H_6_O_4_	35.27	241.5	BQL	BQL
Caffeic acid	C_9_H_8_O_4_	36.14	304.2	BQL	13.08 ± 0.01
Syringaldehyde	C_9_H_10_O_4_	42.62	310.0	BQL	BQL
Ferulic acid	C_10_H_10_O_4_	48.49	318.6	0.52 ± 0.04	1.69 ± 0.02

Abb.: Retention time, *t*_R_; UV maximum absorbance, λ_max_; Non-detected, n.d.; Below quantification limit, BQL.

**Table 6:** LC-ESI-UHR-QqTOF-MS data of phenolic compounds and other metabolites in fresh by-products pineapple liquid fraction. Identification and quantification of the predicted compounds by UPLC analysis.

No.	λ_max_ (nm)	*t*_R_ (min)	^a^*m/z* [M − H]^−^	MS/MS Fragments (*m/z*, % Base Peak Intensity)	Proposed Structure	Ion Elemental Formula	Stem Juice (mg/L)	Peel Juice (mg/L)
1	277	2.6	187.0966 (187.0976)	(187): 143(61), 126(14)	L-Tyrosine	C_9_H_11_NO_3_	---	---
2	278	4.1	315.1089 (315.1085)	(315): 284(9), 153(10)	3,4-Dimethoxyphenyl β-D-glucoside	C_14_H_20_O_8_	---	---
3	295, sh327	4.3	371.0622 (n.d.)	(371): 209(100), 191(20), 147(3), 85(4)	Caffeoyl aldarate	n.d.	---	---
4	266	4.5	293.1242 (293.1249)	(293): 251(15), 119(40), 101(16)	6-O-(2-Hydroxyhexanoyl)-D-glucopyranose	C_12_H_22_O_8_	---	---
5	280	5.2	203.0828 (203.0826)	(203): 186(5), 159(20), 142(29), 116(72)	_L_-Tryptophan	C_11_H_12_N_2_O_2_		
6	271	5.5	360.0940 (n.d.)	(360): 267(10), 249(77), 184(12), 110(77)	1-(1H-Pyrrole-2-carboxyl)-glucuronosyl glycerol	n.d.	---	---
7	296, sh327	6.6	385.2556 (n.d.)	(385): 209(34), 191(100), 147(46), 129(22)	Feruloyl aldarate	n.d.	---	---
8	323	7.5	179.0353 (179.0350)	(179): 135(100)	Caffeic acid	C_9_H_8_O_4_	8.56 ± 0.40	54.12 ± 0.45
9	270	7.8	243.1717 (243.1714)	(243): 199(42), 130(31)	6-[(6-Aminohexanoyl)amino]hexanoate (1)	C_12_H_24_N_2_O_3_	---	---
10	329	7.9	385.1137 (385.1140)	(385): 247(3), 223(13), 205(100)	Sinapoyl hexoside	C_12_H_22_O_10_	---	---
11	278	8.0	205.0509(205.0509)	(205): 119(8)	Eugenin	C_11_H_10_O_4_	---	---
12	299, sh329	8.3	353.0518 (353.0514)	(353): 191(67), 173(100), 155(40), 129(14), 111(93), 85(6)	2-Caffeoylisocitrate	C_15_H_14_O_10_	---	---
13	270	8.5	243.1720 (243.1714)	(243): 199(45), 157(10), 130(36)	6-[(6-Aminohexanoyl)amino]hexanoate (2)	C_12_H_24_N_2_O_3_	---	---
14	278	9.4	371.099 (n.d.)	(371): 249(100), 175(5), 121(77)	n.d.	n.d.	---	---
15	310	9.8	163.0397 (163.0401)	(163): 119(100)	Coumaric acid	C_9_H_8_O_3_	2.36 ± 0.36	8.70 ± 0.51
16	288, sh303	10.2	468.1442 (n.d.)	(468): 306(100), 288(9), 272(39), 254(21), 210(14), 143(35)	S-Coniferylglutathione	C_20_H_27_N_3_O_8_S	---	---
17	275	10.7	441.1343 (n.d.)	(441): 249(100), 225(15), 171(91), 153(7), 128(79)	N-L-γ-glutamyl-*S*-sinapyl-L-cysteine	C_19_H_26_N_2_O_8_S	---	---
18	322	11.0	193.0512 (193.0506)	(193): 178(72), 134(100)	Ferulic acid	C_10_H_9_O_4_	3.02 ± 0.47	11.26 ± 0.66
19	317	12.4	376.2240 (376.2242)	(376): 228(66), 211(13), 164(89)	N-[(Benzyloxy)carbonyl]leucyileucinamide	C_20_H_31_N_3_O_4_	---	---

Abb.: Retention time, tR; UV maximum absorbance, λmax; shoulder, sh; Non-detected, n.d.; a *m/z* [M − H]^−^: experimental values (calculated values). Steingass, et al. [40].
